# Acute Hyperhemolysis Syndrome in a Patient with Known Sickle Cell Anemia Refractory to Steroids and IVIG Treated with Tocilizumab and Erythropoietin: A Case Report and Review of Literature

**DOI:** 10.3390/hematolrep14030032

**Published:** 2022-07-21

**Authors:** Sasmith R. Menakuru, Adelina Priscu, Vijaypal Dhillon, Ahmed Salih

**Affiliations:** Internal Medicine, Indiana University Health, Ball Memorial Hospital, Muncie, IN 47303, USA; apriscu@iuhealth.org (A.P.); vdhillon@iuhealth.org (V.D.); asalih@iuhealth.org (A.S.)

**Keywords:** hyperhemolysis, sickle cell anemia, tocilizumab

## Abstract

Patients with sickle cell anemia often receive multiple red blood cell (RBC) transfusions during their lifetime. Hyperhemolysis is a life-threatening phenomenon of accelerated hemolysis and worsening anemia that occurs when both transfused RBCs and autologous RBCs are destroyed. The level of hemoglobin post-transfusion is lower than pre-transfusion levels, and patients are usually hemodynamically unstable. Hyperhemolysis must be differentiated from a delayed hemolytic transfusion reaction during which destruction of transfused RBC is the cause of anemia. Hyperhemolysis syndrome can be differentiated into acute (within seven days) and chronic forms (after seven days) post-transfusion. The authors present a case of acute hyperhemolysis syndrome in a patient with sickle cell anemia refractory to steroids and IVIG, which are the treatment of choice. The patient was treated with tocilizumab, combined with supportive measures of erythropoietin, iron, vitamin B12, and folate.

## 1. Introduction

Patients suffering from sickle cell anemia, a genetic disorder denoted by homozygous hemoglobin S, have chronic hemolytic anemia and painful episodes that may require blood transfusions to manage complications [1]. Hyperhemolysis syndrome (HHS) is an uncommon but potentially devastating complication of a blood transfusion that can occur in patients suffering from sickle cell anemia in whom pre-transfusion hemoglobin levels are higher than post-transfusion [2]. Patients with HHS need to be differentiated from delayed hemolytic transfusion reactions (DHTR), as those with HHS do not have alloantibodies, and a direct antiglobulin test will be negative [3]. The diagnosis of HHS is difficult, as a high degree of clinical suspicion is needed, but is supported by reticulocytopenia and hyperferritinemia. The pathogenesis of HHS is unknown, but it is postulated that it could be due to a mechanism similar to bystander hemolysis, suppression of erythropoiesis, and RBCs being destroyed by activated macrophages [4]. As acute HHS occurs mainly in SCD patients, a pro-inflammatory state, macrophages are thought to destroy both transfused and autologous RBCs due to already high circulating levels of inflammatory cytokines. Transfusions in a patient suffering from HHS can worsen hemolysis and lead to hemodynamic instability [5]. Management of HHS typically includes glucocorticoids and intravenous immunoglobulin (IVIG), but refractory cases can be treated with either tocilizumab, eculizumab, rituximab, or exchange transfusions [6]. The authors present a case of acute hyperhemolysis syndrome in an African American female treated with the monoclonal antibody tocilizumab and supportive measures.

## 2. Case Report

A 21-year-old African American female with a past medical history of homozygous sickle cell disease presented to the emergency room with complaints of generalized pain, fever, fatigue, and dyspnea for three days. She reported severe pain in her thighs bilaterally but denied any chest pain, shortness of breath, or other associated symptoms. She was taking her prescribed oxycodone-acetaminophen, but she said that the pain became unbearable and thus came to the hospital. The patient was recently discharged from the hospital five days prior for similar symptoms and was treated with pain management, IV hydration, and 1 unit of packed red blood cells due to hemoglobin of 6.7 g/dL. During the current admission, her urine was dark, but she denied any hematuria or dysuria. She was febrile to 38.2 degrees Celsius, tachycardic at 111 beats per minute, and saturating at 97 percent oxygen on room air. On examination, she had conjunctival pallor, skin tenting, and tenderness on palpation of her bilateral thighs; however, all other examination findings were within normal limits. Blood and urine cultures were obtained prior to administering vancomycin and piperacillin-tazobactam. The patient was admitted for sickle cell crisis and was treated with intravenous hydration using normal saline and pain control with hydromorphone. 

Initial workup included a chest X-ray, EKG, CT abdomen and pelvis with IV contrast; blood work included a comprehensive metabolic panel and complete blood count. Pertinent labs showed hemoglobin of 5.3 g/dL (12–15), hematocrit of 19.7% (35–49), white blood cell (WBC) count of 17.2 k/cumm (3.6–10.6), total bilirubin of 5.2 mg/dL (0–1.0), and an LDH of 934 U/L (140–271). All other values were within her normal limits, including WBC count.

Chest X-ray, EKG, and CT abdomen and pelvis did not show any acute findings. Due to her anemia, two units of packed red blood cells were given. Since there was a high suspicion of an aplastic crisis, parvovirus IgG and IgM titers were also obtained. At the time of her blood transfusion, she did not report any adverse effects. However, on repeat laboratory work post-transfusion, her hemoglobin had dropped to 4.5 g/dL. Because she did not respond adequately to transfusion, hematology was consulted, and further laboratory studies were obtained, including a repeat hemoglobin level, direct antiglobulin test (DAT), C-reactive protein (CRP), antibody screen, reticulocyte count, serum bilirubin, ferritin, LDH, and hemoglobin electrophoresis were ordered. DAT was negative, alloantibodies were negative, LDH was elevated to 4230 U/L (135–214), indirect bilirubin was 2.9 g/dL (0.3–1), ferritin was 4327 ng/dL (11–307), CRP was 6.4 mg/dL (<1), and her reticulocyte count was 0.5% (0.5–2.5) despite hemolysis. Hemoglobin electrophoresis showed 45.2% HbS and 44.8% HbA, which indicated an equal ratio of the destruction of hemoglobin, directing our suspicion to an acute hyperhemolysis syndrome. Blood and urine cultures were negative, allowing antibiotics to be discontinued, and parvovirus titers were negative. Of note, she had been following up with a hematologist for her sickle cell anemia, and her disease was well controlled until recently. 

Due to increased levels of LDH, ferritin, and indirect bilirubin coupled with a decreased hemoglobin level and low reticulocyte count and the ratio of HbS to HbA being the same, blood transfusions were avoided, as there was a suspicion for HHS. She was treated with 0.5 g per kilogram of intravenous immunoglobulin and 4 mg per kilogram of prednisone for four days. However, despite treatment, her hemoglobin further decreased to 3.9 g/dL. The patient was put on BiPAP, as she was not maintaining her oxygenation on room air. Amounts of 4000 IU of erythropoietin, IV folate, IV iron, and IV vitamin B12 were also given to support erythropoiesis. As the patient was not improving, salvage therapy with tocilizumab, a monoclonal antibody against IL-6, was given, as there have been reports proving its efficacy in refractory cases. The patient’s hemoglobin slowly improved over her two weeks at the hospital, and at discharge, her hemoglobin stabilized around 8.3 g/dL. Her reticulocyte count was 2%, and her LDH, CRP, ferritin, and indirect bilirubin returned to her baseline. She was told to follow up closely with her hematologist and to immediately return to the emergency department if she experienced similar symptoms.

## 3. Discussion

Hyperhemolysis syndrome is an atypical hemolytic transfusion reaction characterized by a drop in hemoglobin below pre-transfusion levels after transfusing red blood cells [7]. In HHS patients, destruction of both transfused and autologous red blood cells occurs. HHS may develop in pediatric and adult patients suffering from sickle cell anemia, but it may also occur in patients with beta-thalassemia major, myelofibrosis, and anemia of chronic disease [8,9]. The severity and rapid deterioration of this condition require a high degree of clinical suspicion from physicians. The features of HHS in sickle cell patients include fever, pain crises, development of severe anemia, laboratory evidence of hemolysis (hemoglobinuria, hyperbilirubinemia, and elevated LDH), reticulocytopenia, and hyperferritinemia. Further transfusions may precipitate further hemolysis, and recovery is heralded by a rise in hemoglobin and reticulocyte count, with ferritin levels returning to baseline [4].

Hyperhemolysis syndrome must be differentiated from a delayed hemolytic transfusion reaction, as both are distinct clinical entities (with HHS being more severe than DHTR). HHS occurs when both transfused and allogeneic red blood cells are destroyed; in contrast, DHTR occurs when only transfused red blood cells are destroyed by alloantibody formation. This diagnosis of HHS can be made when the ratios of HbS and HbA both decrease concurrently after a transfusion, indicating lysis of autologous and transfused red blood cells. In DHTR, only HbA falls, indicating destruction of transfused red blood cells [10]. HHS can be further divided into acute and delayed forms, where the acute variant occurs within seven days of transfusion, and the chronic variant occurs after seven days [11]. The acute and delayed forms can further be differentiated by direct antiglobulin testing and alloantibody testing. Acute forms have a negative DAT, and alloantibody testing is negative; therefore, as there is no evidence of antibody-mediated destruction, it is postulated that the acute form is macrophage induced [4]. In contrast, in the delayed form of HHS, the DAT is positive, and alloantibodies are often present; therefore, the postulated theory is that transfused RBCs are initially destroyed by antigen/antibody-mediated hemolysis. Afterward, macrophages are recruited by releasing cytokines, such as IL-1 and IL-6, which causes the destruction of autologous RBCs by the mechanism of adhesion [4,12]. In the case presented above, the patient’s hemoglobin was 5.3 g/dL on admission, and after the transfusion of 2 units of blood, her hemoglobin dropped to 4.5 g/dL, which is typical for cases of HHS. 

The mechanism of hyperhemolysis syndrome is thought to be due to changes in the red blood cell membrane coupled with immunological reactions against the exposed membrane phospholipids [10]. There has been no definitive scientifically proven mechanism; the proposed theories include a similar mechanism as bystander hemolysis, suppression of erythropoiesis, and RBC destruction by activated macrophages [4]. According to King et al. and Garraty, bystander hemolysis, a form of delayed hemolytic reaction, occurs when the complement is activated against transfused foreign antigens and the patient’s own cells in the absence of RBC alloantibodies in patients with multiple transfusions [7,13]. Perez et al. suggested that the transfusion-induced suppression of erythropoiesis may cause the increased rate of hemolysis of autologous RBCs [14]. In sickle cell disease, macrophages are already activated, as it is a pro-inflammatory state, and it is theorized that during HHS, the destruction of both RBCs and reticulocytes occurs due to hyperactivity of macrophages because both sickled RBCs and reticulocytes adhere more readily to macrophages, leading to reticulocytopenia and anemia [4]. In most instances of hemolysis, reticulocytosis is common, but a well-documented differential finding in HHS is the presence of reticulocytopenia [15]. During phagocytosis of erythrocytes by macrophages, ferritin is released, resulting in hyperferritinemia in the acute stage of HHS. Therefore, the diagnosis of HHS is further supported by reticulocytopenia and hyperferritinemia, with the improvement of both counts to their normal range after treatment [16]. 

The diagnosis of HHS needs a high degree of clinical suspicion. The pertinent laboratory testing includes direct antibody testing, antibody screening, the ratio of HbA to HbS via either hemoglobin electrophoresis or high-performance liquid chromatography, reticulocyte count compared to baseline, serum bilirubin, serum ferritin, and serum LDH [15]. The etiology of HHS is varied and includes genetics, previous transfusions, acute chest syndrome, and infections [10]. HHS is seen most commonly in patients with SCD, which is likely due to its pro-inflammatory state. Patients with SCD have high levels of circulating cytokines, such as IL-1, TNF-α, and IL-6, which are known to increase the level of macrophages. The authors postulate that measuring cytokine levels and inflammatory markers may be warranted in refractory cases of HHS, as testing becomes more readily available. This would be useful clinically, as it allows for ability to fine tune treatment to block specific cytokines from the activated macrophages. 

The treatment of HHS includes avoiding blood transfusions, as subsequent transfusions can precipitate further acceleration of hemolysis and lead to life-threatening anemia. Therefore, transfusions must be stopped at the first clinical sign of HHS. However, if it is clinically necessary to transfuse blood, then antigenically matched red cells should be utilized. Extended antigen typing must be utilized, including C/c, E/e, K, Jka/ Jkb, Fya/Fyb, M/N, and S/s [14]. In certain cases of severe anemia, exchange transfusions may be useful as well [17]. The mainstay of treatment is glucocorticoids and intravenous immunoglobulin, as they are thought to suppress macrophage activation and help shorten the duration of hemolysis [18]. Erythropoietin has also been utilized in the management of HHS, as it is thought to stimulate erythropoiesis [10]. Rituximab, a chimeric monoclonal antibody against CD20, has been shown to be effective in promoting reticulocytosis [19]. Eculizumab, a recombinant monoclonal antibody against C5, works by stabilizing the red cell membrane, which limits cell lysis [20]. However, rituximab and eculizumab have shown limited effectiveness in treating HHS and DHTR by the National Health Service of England [21].

Tocilizumab, a monoclonal antibody against IL-6, exerts its effect by binding to IL-6R and preventing IL-6 from producing its pro-inflammatory effect. As tocilizumab has been found to be effective in the acute form of HHS, the theory of macrophage activation is further supported [22]. Tocilizumab is currently approved for treating autoimmune conditions, such as rheumatoid arthritis, but has also been utilized in cases of COVID-19. There have been multiple case reports that have shown tocilizumab to be effective in treating HHS [22,23,24]. In each of the cases, including ours, there have been no reported complications to tocilizumab, with rapid clinical improvement. In refractory cases of HHS, tocilizumab may be warranted in the management.

## 4. Conclusions

This case is essential in the current literature, as it describes the uncommon phenomenon of HHS and the methods required to diagnose the condition. Physicians need a high degree of clinical suspicion in cases of HHS, as further blood transfusions may lead to life-threatening anemia. The treatment typically utilizes IVIG and steroids to blunt the activation of macrophages; however, in rare instances, such as in this case, HHS may be refractory. Utilizing tocilizumab, an IL-6 inhibitor, in SCD-induced acute HHS may be warranted, given that the mechanism of the syndrome is macrophage driven and that SCD itself is a pro-inflammatory state. The authors recommend more research on the correlation between cytokine levels and HHS, as targeted therapy may be possible once testing becomes more readily available. 

## Data Availability

Not applicable.
